# Anesthetic Management and Considerations for Cesarean Delivery in a Patient With Cystic Fibrosis

**DOI:** 10.1155/cria/6388254

**Published:** 2025-01-21

**Authors:** Richard Pham, Chris Mehdizadeh, Christopher Baker, Leonard J. Soloniuk, Ioana Pasca, Ashish Sinha

**Affiliations:** ^1^Department of Medicine, University of California Riverside School of Medicine, Riverside, California, USA; ^2^Department of Anesthesiology and Perioperative Medicine, Riverside University Health System, Moreno Valley, California, USA; ^3^Department of Medicine, Philadelphia College of Osteopathic Medicine-South Georgia, Moultrie, Georgia, USA; ^4^Department of Gynecology and Obstetrics, Loma Linda University School of Medicine, Loma Linda, California, USA; ^5^Department of Anesthesiology, Loma Linda University School of Medicine, Loma Linda, California, USA

## Abstract

Recent advancements in therapeutics and risk reduction in the management of cystic fibrosis have increased the life expectancy of cystic fibrosis patients to the fifth decade of life. As the life expectancy of cystic fibrosis patients has increased, more cystic fibrosis patients have opted to pursue pregnancy. Normal pregnancy is accompanied by physiological changes that affect anesthetic management. These normal physiological changes, combined with the pathological manifestations of cystic fibrosis, create a unique set of anesthetic challenges. Here, we report on the management and clinical course of a 37-year-old parturient with cystic fibrosis, focusing on the anesthetic approach.

## 1. Introduction

Cystic fibrosis (CF), caused by genetic mutations in the cystic fibrosis transmembrane conductance regulator (CFTR) protein, affects over 30,000 individuals in the United States [1]. Mutations in the *CFTR* gene lead to defective chloride transport resulting in thick, sticky mucus production in various organs including the lungs, pancreas, liver, intestines, and sweat glands [2]. The abnormal mucus in the lungs obstructs airways predisposing individuals to recurrent lung infections, chronic inflammation, and progressive lung damage. In the pancreas, the mucus disrupts the release of digestive enzymes which results in impaired nutrient absorption, frequently leading to malnutrition. Advances in understanding the genetics and pathophysiology of CF have led to improved diagnostic techniques and therapeutic interventions. Although with more than 500 different mutations, only the most common mutations are tested for, making the late detection of CF more common later in life if a rare mutation is present [3].

During the course of pregnancy, numerous physiological changes occur to support the developing fetus and ensure maternal well-being. In addition to these normal physiological changes, the pathological pulmonary and extra-pulmonary manifestations of CF include pancreatic exocrine insufficiency, CF-related diabetes, reduced bone density, distal intestinal obstruction, gastroesophageal reflux disease (GERD), and difficult vascular access, all of which may complicate anesthetic management of these patients [4]. A Health Insurance Portability and Accountability Act (HIPAA) authorization was obtained for the publication of this case report.

## 2. Case Description

A 37-year-old female, G2P0010, with a history of endometriosis and CF, was admitted for fetal growth restriction at 32 weeks' gestation. Umbilical artery Doppler studies revealed intermittently absent end-diastolic flow. Betamethasone was administered for fetal lung maturation, and a cesarean delivery was scheduled, in two weeks at 34 weeks gestation. Prior to admission, the patient reported frequent headaches, vision changes with scotomas for the past week, and worsening hyperemesis gravidarum, especially at night with obstetric provider concerns for preeclampsia. She remained normotensive throughout her hospital admission and preoperative period.

She had a history of recurrent, severe lung infections starting at around the age of 13, necessitating IV antibiotic therapy and multiple hospitalizations for pneumonia. Two years before her current hospital admission, she was hospitalized for coronavirus disease (COVID-19) pneumonia, requiring mechanical ventilation. Post-discharge genetic testing revealed CF due to 1 copy of the *A455E* mutation. This mutation enables the normal functioning of the *CFTR* protein but causes the transmembrane protein to be produced in insufficient quantities due to a shortened half-life of the protein. Pulmonary function tests confirmed restrictive lung disease, but the numeric results were not available to her clinical caregivers at the time of this admission and delivery. Since the COVID-19 hospitalization, she has experienced chronic dyspnea on exertion and productive cough with intermittent thick, yellow/brown sputum. In her third trimester, she developed shortness of breath at rest and her SpO_2_ dipped to 80%–85% with ambulation. The patient was not on supplemental oxygen at home. She resides in a town which is at an elevation of approximately 5400 ft (1650 m) above sea level. She denied any smoking history.

Prior to her procedure, a preoperative electrocardiogram and an echocardiogram were performed to evaluate pulmonary and cardiac status. The electrocardiogram and echocardiogram were unremarkable and notably showed no evidence of elevated pulmonary artery pressures. The patient was also administered nebulized albuterol sulfate and ipratropium bromide every 12 h and as needed in the days prior to her surgical delivery. Her airway was assessed to be Mallampati class I and her risk stratification was deemed to be an American Society of Anesthesiologists physical status 3.

A spinal anesthetic technique was chosen for this primary cesarean delivery since prolonged operative times were not anticipated. The patient was administered an uneventful subarachnoid block performed in the sitting position at L2-3. The block was assessed at the T6 level prior to incision, exactly as planned by the anesthesia team. Phenylephrine boluses were administered as needed to maintain an appropriate blood pressure but were not required after delivery. After delivery, the obstetrician reported a satisfactory uterine tone as achieved by the oxytocin infusion started after the delivery of the placenta. The procedure was overall tolerated well without any complications. The total anesthesia time was 105 min and the patient was transferred to the Labor and Delivery recovery unit with subsequent routine postoperative care. Neither ICU level of care nor supplemental oxygen on the floor was required during the course of her admission. She did not require antibiotic therapy to treat pulmonary infections. There was no evidence on examination or laboratory testing for malnutrition. Postoperatively, the patient and the baby were stable. The patient was discharged home on postoperative day 3.

## 3. Discussion

The life expectancy for individuals born with CF, over the past 70 years, has increased substantially. The estimated median survival age was 4–5 years in 1954 and increased to approximately 48 years in 2019 [5, 6]. With improvements in management, there has been a significant secular increase in CF pregnancies with nearly 80% of females with CF intending to pursue pregnancy [7]. Research indicates that CF pregnancies tend to occur at a younger age compared to non-CF pregnancies, possibly reflecting a proactive approach to family planning before an anticipated decline in pulmonary function [8]. Additionally, assisted reproductive techniques are more frequently utilized in CF pregnancies, likely due to infertility issues associated with this condition [8]. Women with CF have a higher likelihood of experiencing multiple gestation, assisted vaginal delivery, gestational diabetes, GERD, and preterm labor compared to those without CF [2]. However, there is no significant difference in the odds of cesarean delivery, hypertensive disorders of pregnancy, placental abruption, fetal growth restriction, and chorioamnionitis [2]. Nevertheless, pregnancies in CF patients carry an increased risk of mortality (OR 125 (95% CI 67–233); *p* < 0.0001) and blood transfusion at delivery (OR 1.7 (95% CI 1.1–2.7); *p*=0.01), as well as increased risk of respiratory complications such as pneumonia (OR 68.7 (95% CI 54.3–86.9); *p* < 0.0001), mechanical ventilation requirement (OR 31.9 (95% CI 21.4–47.5); *p* < 0.0001), and acute respiratory failure (OR 29.6 (95% CI 16.7–48.0); *p* < 0.0001) [2].

Predicting outcomes in CF pregnancies and anticipating associated pathologies are crucial for effective management and care. CF patients with FEV_1_ > 60% typically experience favorable outcomes during pregnancy [4]. Conversely, a pre-pregnancy FEV_1_ of less than 60% is linked to a higher incidence of maternal complications, including pulmonary exacerbations, pneumothorax, and preeclampsia [4]. Pulmonary infections, particularly those caused by *Burkholderia cenocepacia*, are also associated with rapid declines in lung function and should be considered in management of CF pregnancies [9]. Furthermore, women with CF often have comorbidities such as asthma, diabetes mellitus, cardiac conduction disorders, and thrombophilia [2]. Pre-existing cor pulmonale and severe pulmonary hypertension are considered contraindications to pregnancy. CF pregnancies complicated by primary pulmonary arterial hypertension demonstrate high maternal mortality rates [10]. These predictive factors and associated pathologies underscore the importance of comprehensive assessment and individualized management strategies for pregnant patients with CF.

Managing CF in pregnancy involves a multidisciplinary approach aimed at optimizing maternal and fetal outcomes [11]. Management typically focuses on maintaining respiratory function, optimizing nutritional status, and addressing potential complications. During pregnancy, the increased oxygen demand and changes in respiratory mechanics may exacerbate breathing difficulties in individuals with CF, especially if they already have compromised pulmonary function [4, 12]. Moreover, hormonal changes can affect mucus production, potentially worsening respiratory symptoms. Respiratory therapies, such as chest physiotherapy, bronchodilators, and inhaled medications are crucial for managing airway clearance and preventing exacerbations. Antibiotic therapy may be necessary to treat respiratory infections promptly. Additionally, CF-related pancreatic insufficiency may pose challenges in meeting the increased nutritional demands of pregnancy, potentially leading to malnutrition or inadequate weight gain. Nutritional support, including enzyme replacement therapy and dietary adjustments, aims to ensure adequate weight gain. Regular monitoring of lung function, nutritional status, and fetal growth is essential for early detection of complications. In some cases, hospitalization may be necessary for intensive treatment and monitoring, especially during acute exacerbations or delivery.

CF patients often experience obstructive respiratory issues such as air trapping and higher intrinsic positive end-expiratory pressure (PEEP), which can lead to complications like barotrauma, bronchospasm, and pneumothorax with mechanical ventilation. Neuraxial techniques are generally preferred over the use of general anesthesia. General anesthesia, with its manipulation and instrumentation, poses a higher risk of exacerbating respiratory problems, potentially delaying extubation at the end of cesarean delivery [13]. Pain control is crucial during the peripartum period to ensure patients can continue chest physical therapy, clearing secretions by coughing, maintaining respiratory health, and early mobilization. Using a multimodal pain management approach helps reduce the risk of opioid-induced respiratory depression. Routine CF pulmonary care should be continued, including treatment with inhalers and mucolytics as well as continuation of chest physiotherapy.

Neuraxial anesthesia is the preferred anesthetic for cesarean deliveries. A spinal anesthetic technique was chosen for this primary cesarean delivery since prolonged operative times were not anticipated. Some may consider an epidural or a combined spinal–epidural (CSE) technique over a subarachnoid anesthetic. Since avoiding endotracheal intubation and general anesthesia is a paramount concern, it may be that a CSE would have been preferable for this patient, since it allows for slower onset and titration and mitigating the risk of a high spinal block. However, the data supporting this recommendation are considered low quality and based primarily on expert opinion [14].

The presence of CF in parturients necessitates the need for careful evaluation, close monitoring, and specialized care for pregnant women with CF to optimize maternal and fetal outcomes.

## Data Availability

Data sharing is not applicable to this article as no new data were created or analyzed in this study.

## Nomenclature

CF: Cystic fibrosis
CFTR: Cystic fibrosis transmembrane conductance regulator protein
COVID-19: Coronavirus disease
FEV_1_: Forced expiratory volume in one second
GERD: Gastroesophageal reflux disease
CSE: Combined spinal–epidural
